# Thermosensitive Hydrogels Loaded with Resveratrol Nanoemulsion: Formulation Optimization by Central Composite Design and Evaluation in MCF-7 Human Breast Cancer Cell Lines

**DOI:** 10.3390/gels8070450

**Published:** 2022-07-19

**Authors:** Sabna Kotta, Hibah Mubarak Aldawsari, Shaimaa M. Badr-Eldin, Anroop B. Nair, Mohammed Kaleem, Mahmood Hassan Dalhat

**Affiliations:** 1 Department of Pharmaceutics, Faculty of Pharmacy, King Abdulaziz University, Jeddah 21589, Saudi Arabia; haldosari@kau.edu.sa (H.M.A.); smbali@kau.edu.sa (S.M.B.-E.); 2 Center of Excellence for Drug Research and Pharmaceutical Industries, King Abdulaziz University, Jeddah 21589, Saudi Arabia; 3 Department of Pharmaceutical Sciences, College of Clinical Pharmacy, King Faisal University, Al-Ahsa 31982, Saudi Arabia; anair@kfu.edu.sa; 4 Department of Biochemistry, Faculty of Science, King Abdulaziz University, Jeddah 21589, Saudi Arabia; kaleemmubin88@gmail.com (M.K.); mdalhatdalhat@stu.kau.edu.sa (M.H.D.); 5 Department of Pharmacology, Faculty of Pharmacy, Dadasaheb Balpande College of Pharmacy, Nagpur 440034, India

**Keywords:** thermosensitive hydrogel, rheology, breast cancer, nanoemulsion, resveratrol, design of experiments, cell lines

## Abstract

The second most common cause of mortality among women is breast cancer. A variety of natural compounds have been demonstrated to be beneficial in the management of various malignancies. Resveratrol is a promising anticancer polyphenolic compound found in grapes, berries, etc. Nevertheless, its low solubility, and hence its low bioavailability, restrict its therapeutic potential. Therefore, in our study, we developed a thermosensitive hydrogel formulation loaded with resveratrol nanoemulsion to enhance its bioavailability. Initially, resveratrol nanoemulsions were formulated and optimized utilizing a central composite-face-centered design. The independent variables for optimization were surfactant level, homogenization speed, and time, while the size and zeta potential were the dependent variables. The optimized nanoemulsion formulation was converted into a sensitive hydrogel using poloxamer 407. Rheological studies proved the formation of gel consistency at physiological temperature. Drug loading efficiency and in vitro drug release from gels were also analyzed. The drug release mechanisms from the gels were assessed using various mathematical models. The effect of the optimized thermosensitive resveratrol nanoemulsion hydrogel on the viability of human breast cancer cells was tested using MCF-7 cancer cell lines. The globule size of the selected formulation was 111.54 ± 4.16 nm, with a zeta potential of 40.96 ± 3.1 mV. Within 6 h, the in vitro release profile demonstrated a release rate of 80%. According to cell line studies, the produced hydrogel of resveratrol nanoemulsion was cytotoxic to breast cancer cells. Overall, the results proved the developed nanoemulsion-loaded thermosensitive hydrogel is a promising platform for the effective delivery of resveratrol for the management of breast cancer.

## 1. Introduction

Breast cancer is the most frequent cancer among women, as well as the second most common cancer in the general population. In 2020, almost 2.3 million new cases were reported [1]. Despite the fact that mortality has dropped in recent years, breast cancer remains a major health concern. The clinical improvement of new treatments and also the availability of novel drugs and formulations showed an obvious benefit for those who are in the advanced stages of the disease also [2].

Natural compounds have been extensively researched as a source of uncountable bioactive agents with distinct actions. Out of these, several dietary phytochemicals have been widely investigated for their cytotoxic effects in many cellular models to prove anticancer activity [3]. Among these, resveratrol is a polyphenolic non-flavonoid compound that occurs in nature. The main sources include fruits, such as grapes and mulberries, and also seeds, roots, flowers, grains, vegetables, etc. [4]. It has been extensively studied for its health-promoting advantages, including its anticancer, neuroprotective, anti-inflammatory, antioxidant, and cardio-protective activities [5,6,7,8,9,10]. It is proven that resveratrol can prevent or inhibit cancer at all stages, including initiation, promotion as well as cancer progression. Many studies have demonstrated the anticancer effects of resveratrol along with its cellular mechanisms, as well as its transduction pathways, in vitro and in vivo [11,12,13,14]. Numerous researchers propose that a strong anti-tumor activity is due to induction of apoptosis or cell cycle arrest, or both, in mammary cancer cell lines. Many in vivo animal studies have also proved a reduction in the incidence of mammary tumor formation, as well as delayed tumor onset and progression, due to resveratrol treatment [15,16]. Nevertheless, as a polyphenolic compound, resveratrol is also characterized by poor bioavailability due to limited aqueous solubility. Furthermore, resveratrol goes through a number of first-pass metabolisms, and is quickly removed from the body [17]. All these factors contribute to its poor bioavailability. Therefore, a high dose is essential to achieving therapeutically active levels [18]. As a result, numerous methods for increasing resveratrol bioavailability have been discovered [19,20]. New drug delivery systems using nanotechnology have been widely researched for resveratrol delivery. Polymeric nanomicelles, metal, and polymeric nanoparticles, nanoemulsion, liposomes, etc. are a few among them [21]. Different delivery routes have also been researched [19,22]. Among the different nanotechnology approaches, nanoemulsions have proved to be very effective, due to their advantages, such as extensive surface area and stability, over other nanotechnology systems [23,24]. Recently, the potential of nanoemulsion in breast cancer therapy has also been demonstrated by various researchers [25,26,27,28,29]. Hence, in the current study, we aimed to utilize the advantages of nanoemulsion along with temperature-sensitive hydrogel delivery systems for the management of breast cancer. Tumor responsiveness proved to be better with sustained drug contact with chemotherapeutics [30,31]. Thus, increasing the length of time during which chemotherapeutics are retained within the desired site is favorable for treatment. To increase the drug retention time at the site of application, hydrogels have been widely explored by many scientists [32,33,34]. Hydrogels are a class of substances that are appropriate for various applications in the food, pharmaceutical, and cosmetic industries, due to the characteristics of their solid-like network. The solid network of a hydrogel is formed as a result of a physical, chemical, or a physicochemical assembly of gelators. These hydrogel matrices can encapsulate and release a diverse variety of compounds, and these properties are exploited in a variety of applications [35].

Over the last decade, temperature-responsive gel systems have gained much attention in pharmaceutical applications. These kinds of formulas can undergo sol–gel transitions at physiological temperatures to form a physically cross-linked gel matrix. At room temperature, they possess a low viscosity, and at body temperature, transition to gel occurs, which makes them appropriate for fine spreading. These types of temperature-sensitive formulations are widely used in topical as well as injectable systems for site-specific drug delivery. Thus, after application, these kinds of in situ formulations become physically cross-linked to form a gel-like matrix, so that drug release can be sustained for a long period. Poloxamers have been widely explored in this regard as a temperature-sensitive polymer [36,37,38]. In the current study, we utilized poloxamer hydrogels loaded with nanoemulsion formulation of resveratrol to enhance the bioavailability by increasing the contact time, as well as reducing the particle size.

The main objective of this investigation was to develop a thermosensitive hydrogel nano formulation loaded with resveratrol to enhance the bioavailability. In addition, we investigated how well the high-speed homogenization technique worked in the creation of resveratrol nanoemulsions. The optimization process was achieved by central composite design. The size and charge of the nanoemulsion were selected as variables for formulation optimization. The effect of time, speed of homogenization, and percentage of surfactant were studied as independent factors. Coconut oil was chosen for the oil phase, and the surfactant was sodium caseinate. The optimized nanoemulsion was converted to hydrogels using poloxamer 407, and drug loading efficiency, as well as the in vitro drug release profile of the formulation, were evaluated. The mechanism of drug release was also evaluated. The efficiency of the optimized hydrogels formulation was studied in MCF-7 human breast cancer cell lines. To our knowledge, no research on hydrogels of resveratrol nanoemulsion was explored for the treatment of breast cancer. Since there has been a significant focus in recent years on advances in nanohydrogel-based local therapy for chemotherapy [39], this strategy is a promising solution for the management of breast cancer.

## 2. Materials and Methods

### 2.1. Materials

Resveratrol, poloxamer 407 and sodium caseinate were purchased from Sigma Aldrich, Darmstadt, Germany. Double-filtered pure coconut oil was purchased from KERA^®^, KERAFED, Thiruvananthapuram, India. All the other reagents used were of analytical grade.

### 2.2. Preparation and Optimization of Resveratrol Nanoemulsion

Preparation of nanoemulsions was accomplished via blending surfactant solutions with coconut oil. Sodium caseinate at different concentrations was used as surfactant, and resveratrol-loaded coconut oil (25 mg/mL) was used as the oil phase. Table 1 lists the factors and levels that were chosen.

First, coarse emulsions were created by mixing the drug-loaded oil with surfactant-containing aqueous phase in varying concentrations under magnetic stirring for 5 min. The oil content was kept constant, while surfactant strengths ranged from 2% to 6%. Nanoemulsions were made by adjusting the speed and time of a high-speed homogenizer (ULTRA TURRAX IKA T18 basic), as specified by the experimental design (Table 1).

The central composite design was implemented for formulating and optimizing resveratrol nanoemulsions. Two process variables, homogenization speed (A, rpm) and homogenization time (B, min), and one formulation variable, surfactant concentration (C, %), were investigated as independent variables. The lower and higher levels of the variables are presented in Table 1. Droplet size (nm) and zeta potential (mV) were considered as responses. The software Design Expert (Version 12) was used to generate 19 trial runs, as shown in Table 2. The sequential model fitting to each response was selected as per the highest determination coefficient (R^2^). The responses were analyzed according to the best-fitting model using ANOVA at *p* < 0.05.

### 2.3. Characterization of Optimized Nanoemulsion

#### 2.3.1. Droplet Size, Size Distribution (PDI), and Zeta Potential

The droplet size of formulated nanoemulsions was measured using Zetasizer Nano (Malvern Instruments Limited, Worcestershire, UK). DLS (Dynamic Light Scattering) technique was the principle behind this analysis. Diluted samples (100 times) after sonication were used to analyze the size to prevent multiple scattering effects [40]. All measurements were made three times. PDI gives information about particle size distribution [41]. A lower PDI score indicates that the globule size in the formulation is uniform.

The Zetasizer Nano (Malvern Instruments Limited, Worcestershire, UK) was used to determine the zeta potential of nanoemulsion droplets. The electrokinetic potential in colloidal systems, or the electric potential in the interfacial double layer, is referred to as zeta potential. The potential difference between the dispersion media and the stationary fluid layer coupled to the dispersed particles is measured in [42]. To avoid multiple scattering effects, 100-fold dilution was performed before experimenting. All measurements were made three times.

#### 2.3.2. Surface Morphology by TEM

A TEM image of the optimized nanoemulsion with proper dilution was recorded. Negative staining with 2% phosphotungstic acid was carried out, and the sample was placed over a copper grid, whereupon the images were taken (JEOL, JEM 1010, Tokyo, Japan; 60–80 kV).

#### 2.3.3. Thermodynamic Stability Studies

The thermodynamic stability of the optimized resveratrol nanoemulsion was tested using alternative heating–cooling cycles, freeze–thaw cycles, and centrifugation. For the heating–cooling cycles, the nanoemulsion was subjected to six cycles alternating between −4 °C and 45 °C for 48 h. Freeze–thaw cycles were also carried out on the optimized formulation, alternating between −21 °C and 25 °C for 48 h. The optimized formulation was centrifuged at 3500 rpm for 30 min [43].

### 2.4. Formulation of Nanoemulsion-Loaded Pluronic Hydrogels and Rheological Characterization

The optimized nanoemulsions were converted into temperature-sensitive hydrogels. The weighed quantity of poloxamer 407 (20%) was added to the optimized nanoemulsion formulation and maintained while stirring at room temperature overnight [38].

Brookfield’s viscometer, DV model (Boston, MA, USA), was used to determine the viscosity of the compositions. The viscosity of the nanoemulsion hydrogel formulation was measured at 25 °C and 37 °C. The viscosity was recorded as an average of three readings.

### 2.5. In Vitro Drug Release Study

The release of resveratrol from the optimized hydrogel was compared to the resveratrol suspension. Resveratrol (8.3 mg/mL) was suspended in 1% sodium CMC in water to make a resveratrol suspension. The in vitro release study from the hydrogels as well as resveratrol suspension was investigated using a molecular cut-off 12,000 Da dialysis cellulose membrane (Sigma–Aldrich, St. Louis, MO, USA) in USP type 2 dissolution equipment [44]. The experiment was carried out at 37 °C in 900 mL of pH 7.4 phosphate buffer, with the dissolution medium being agitated at 50 rpm. Formulations equivalent to 25 mg of resveratrol were loaded in the tubing, closed with the use of a clip and tied to the paddle. To quantify the drug release, 5 mL aliquot samples were withdrawn at specified time points and replaced with a new medium to maintain the constant volume. The drug release from the nanoemulsion hydrogels was compared to resveratrol suspension. The samples were quantified for drug content at 306 nm using a UV-VIS spectrophotometer (UV-2600, Shimadzu, Kyoto, Japan).

### 2.6. Drug Release Kinetics

To study the release kinetics, data obtained from the in vitro drug release studies were plotted in various kinetic models. The resveratrol release profile from the gel with respect to time was utilized to calculate the correlation coefficient, r^2^, and kinetics of drug release by different mathematical models [45]. Zero-order, first-order, Higuchi, and Korsmeyer–Peppas models were utilized for examining the drug release kinetics from the hydrogel matrix, with zero-order (Equation (1)) as the cumulative amount of drug released versus time, first-order (Equation (2)) as log cumulative percentage of drug remaining versus time, and Higuchi’s model (Equation (3)) as cumulative percentage of drug released versus square root of time. The best-fitted model corresponded to the one showing a linear regression coefficient close to 1. To evaluate the mechanism of drug release from the optimized hydrogel formulations, data for the first 60% of drug release were plotted in the Korsmeyer–Peppas equation (Equation (4)) as log cumulative percentage of drug released versus log time. The equations for the various mathematical models are given below [46,47].

Zero-order model *Q_t_* = *Q*_0_ + *K*_0_*t* (1)

First-order model ln*Q_t_* = *lnQ*_0_ + *K_t_* (2)

Higuchi model *Q_t_* = *K_h_* × *t*^0.5^ (3)

Korsmeyer–Peppas model *Q_t_* = *K_Kp_t*^n^ (4)

where *Q_t_* denotes the amount of drug released at time *t*, *Q*_0_ denotes the initial amount of drug, *K*_0_ represents the zero-order kinetic constant, *K_t_* represents the first-order rate constant, and *n* denotes the drug release exponent.

### 2.7. Cell Culture

MCF-7 cells were purchased from ATCC (American Type Culture Collection), (Manassas, VA, USA). MCF-7 cells were cultured in DMEM (Dulbecco’s modified Eagle’s medium) (Sigma) and complemented with fetal bovine serum (FBS10%) (Sigma St. Louis, MO), 100 I.U/mL penicillin, and 100 μg/mL streptomycin (Sigma St. Louis, MO). The cells were cultivated at 37 °C and 5% CO_2_ in a humidified incubator.

#### Cell Viability Assay

The cells were planted in 96-well plates and allowed to develop to 70–80% confluence. The MCF7 cells were added at different concentrations (20, 30, and 40 μM) to resveratrol nanoemulsion and resveratrol suspension for 24 h with Dimethyl sulfoxide (DMSO). After draining the medium, each well was filled with sterile (filtered) 3(4,5dimethylthiazol2yl)2,5diphenyltetrazolium bromide (MTT) (10 L; 5 mg/mL in phosphate-buffered saline) to achieve a standard solution of 0.4 mg per mL. Plates were again kept for incubation for 3 h to allow metabolizing of MTT by viable cells to insoluble formazan crystals. The media and unaltered MTT were withdrawn from each well, and 150 µL of DMSO was added to ensure full formazan solubilization. The absorbance was then measured at 490 nm using a BioTek SynergyH1 microplate reader [48,49].

### 2.8. Stability Study

The emulsion formulation technique and formulation parameters highly influence the physicochemical stability of the formulation during storage. A perfect formulation results in stable emulsion over a long period. Variations in particle size, PDI, or zeta potential usually indicate that flocculation or coalescence and Oswald ripening are happening [23]. The two most important mechanisms that destabilize the emulsion and cause variation in droplet size distribution are Ostwald ripening and coalescence [50]. Ostwald ripening mainly occurs due to the difference in solubility between smaller and larger droplets, and also to polydispersity. Coalescence is caused by the rupturing of the outer films of the continuous phase and the fusion of two globules into one larger droplet [51,52]. The stability of the optimized formulation was assessed for 3 months by storing it at room temperature, and the droplet size, polydispersity index, and zeta potential were measured.

### 2.9. Statistical Analysis

The experimental data were examined using one-way analysis of variance (ANOVA) and then the Tukey multiple comparison test in Prism 9 (GraphPad Software, San Diego, CA 92108, USA) to determine statistical significance.

## 3. Results and Discussion

### 3.1. Effect of Variables on Droplet Size

As per the model fit statistical analysis, a quadratic model was suggested for droplet size. The significance of the model is indicated by the Model F-value of 323.22 in the ANOVA analysis, Table 3. There was only a 0.01% chance that this value could be that high by virtue of noise. The observed data demonstrated greater corrected R^2^ (0.9949), anticipated R^2^ (0.9623), and adequate precision (59.0664). The corrected R^2^ and anticipated R^2^ values were close enough for the quadratic model to be considered suitable.

**Figure 1:**
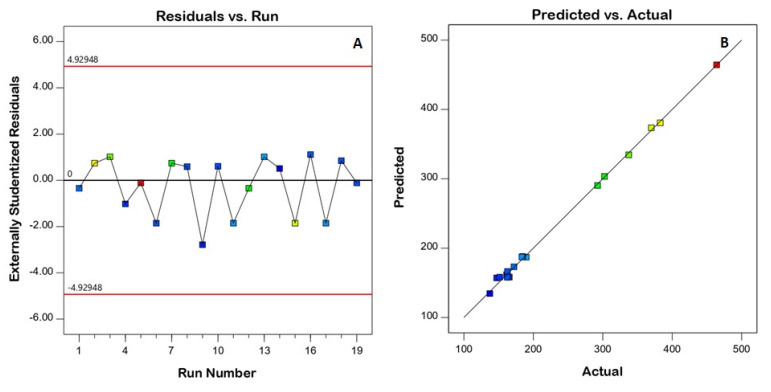
Diagnostic plots for the quadratic models for the droplet size of resveratrol nanoemulsions (**A**) residuals versus the run, (**B**) predicted versus actual values.

To ensure the model’s goodness of fit, diagnostic plots were constructed. The residuals versus run plot presented in Figure 1A highlights that no lurking variable affected the measured response, while the observed versus predicted values plot presented in Figure 1B shows the good agreement between both values, indicating the credibility of the model. The homogenization speed (A), homogenization time (B), and surfactant concentration (C) were shown to have a strong impact on nanoemulsion size (*p* < 0.05), according to the ANOVA results. Detailed ANOVA data are provided in Table 3.

The quadratic polynomial model for globule size data was created using the following equation:

Size = +149.98 − 11.25A (1) 4.13A (2) + 9.08B−71.92C − 51.27A (1)B − 0.8729A (2)B 46.96A (1) C + 10.63A (2) C + 37.37BC + 18.98B^2^ + 122.07C^2^

**Figure 2:**
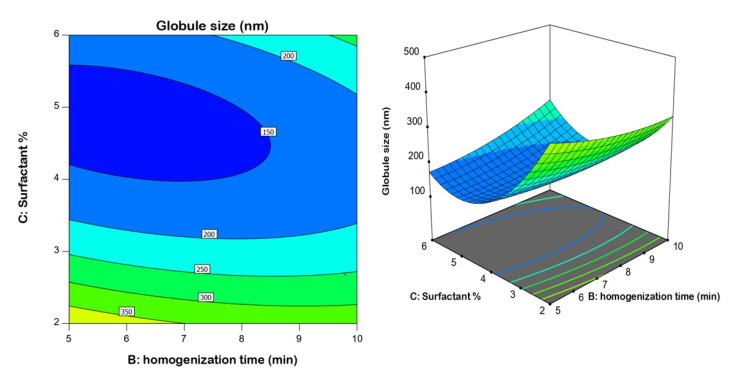
2D contour and 3D response surface plots revealing the effects of surfactant % and homogenization time at an average speed on nanoemulsion droplet size.

The surfactant concentration had the greatest influence on globule size, as demonstrated by its highest coefficient in the derived equation and lowest *p*-value of 0.0001. It was observed that the globule size decreased at higher surfactant concentrations (Figure 2); such an inverse relationship is supported by the negative sign of the term C in the equation. The smaller size could be attributed to the role of surfactant in the creation of nanoemulsions by decreasing the interfacial tension, which lowers the Laplace pressure and lowers the stress needed for droplet deformation. This finding coincides well with previous studies [53,54,55].

### 3.2. Zeta Potential

**Figure 3:**
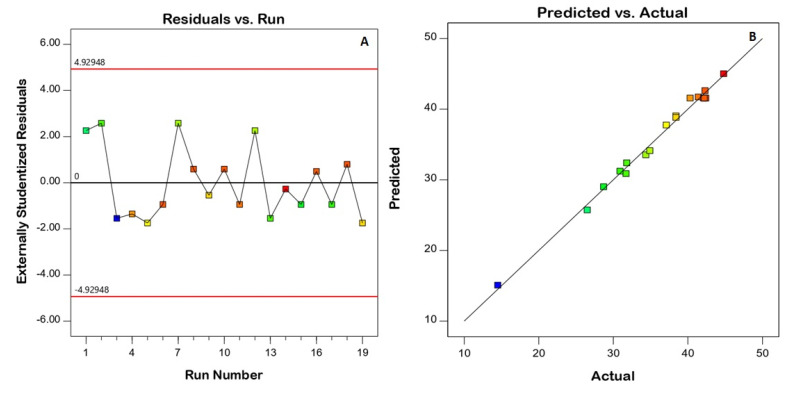
Diagnostic plots for the quadratic model for the zeta potential of resveratrol nanoemulsions. (**A**) Residuals versus the run, (**B**) predicted versus actual values.

**Figure 4:**
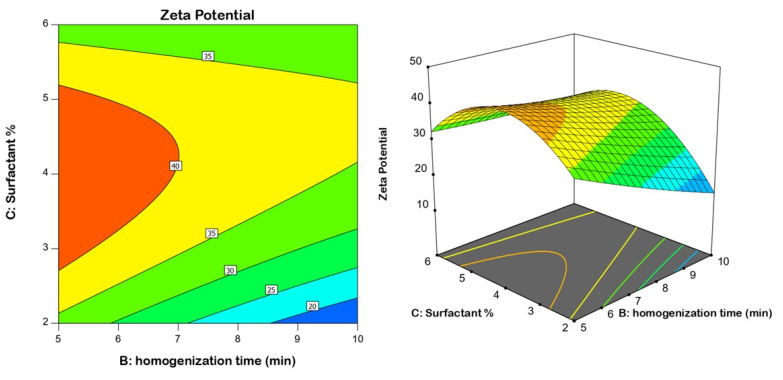
2D contour and 3D response surface plots revealing the effect of surfactant % and homogenization time at an average speed on the nanoemulsion zeta potential.

Model fit statistics revealed the fitting of the zeta potential data to the quadratic model. The significance of the model was supported by the ANOVA F-value of 78.43 (Table 4). There was only a 0.01% probability that this value could be that high by virtue of noise. Corrected R^2^, anticipated R^2^, and adequate precision for the model were found to be 0.9793, 0.7891, and 35.036, respectively. The corrected R^2^ and anticipated R^2^ values were close enough to consider the suggested model. Diagnostic plots were generated to assess the goodness of fit of the suggested model. The residuals versus run plot presented in Figure 3A highlights that no lurking variable affected the measured ZP, while the observed versus predicted values plot presented in Figure 3B reveals good harmony between both values; thus the model credibility is affirmed. The investigated factors, namely, speed of homogenization (A), homogenization time (B), and surfactant concentration (C), were found to have a significant influence on nanoemulsion zeta potential at *p* < 0.05. Figure 4 displays the contour and surface plots for the numerical variables on the zeta potential. Table 4 contains a detailed analysis of variance (ANOVA) data.

The following equation was created to describe the quadratic model for the zeta potential:

Zeta Potential = +41.81 + 3.09A (1) + 0.1164A (2) − 1.01B + 0.9950C + 3.71A (1)B − 0.2775A (2)B − 2.72A (1)C − 0.0525A (2)C + 4.23BC + 0.5992B^2^ − 11.45C

### 3.3. Optimization of Resveratrol Nanoemulsion

The optimum levels of the studied variables were selected based on numerical optimization. The optimized formulation that would achieve minimized size and maximized zeta potential was predicted as follows: a homogenization speed of 14,000, homogenization time of 8.399 min, and a surfactant concentration of 4.028%. The projected globule size of 120.287 nm and zeta potential of 46.965 nm matched the measured size (111.54 2.16 nm) and potential (40.963.1), indicating that the optimization method was successful.

### 3.4. Characterization of Optimized Nanoemulsion

#### 3.4.1. Globule Size and PDI

**Figure 5:**
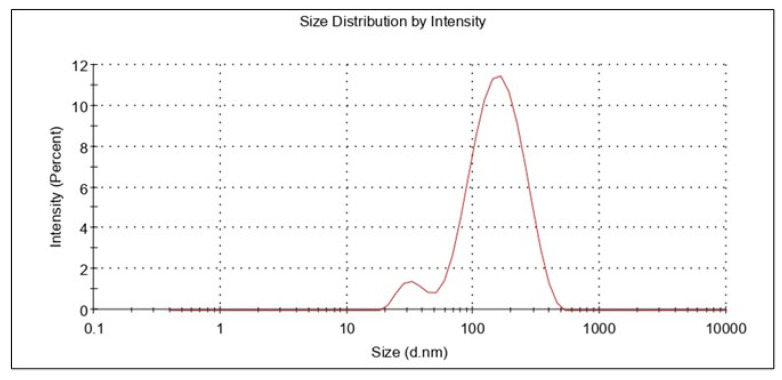
Representative plots for globule size of the developed nanoemulsion.

The information concerning droplet size and distribution is particularly important for achieving nanoemulsion performance, as the size of nanoemulsion droplets affects drug release and absorption. The size of the globules, in addition to their composition, influences the bio-acceptability of the dose form [53,56]. It was noticed that the amount of surfactant used influenced the droplet size of the nanoemulsion. The polydispersity index and droplet size of the optimized nanoemulsion were determined. Polydispersity is measured as the ratio of the mean difference to average globule size, which indicates globule size homogeneity inside the system [57,58]. The smaller the PDI, the higher the uniformity of the globules in the formulation. It was reported that the preferred value was less than 0.5. If the PDI was high, there was a chance of coalescence of the droplets. The mean globule size and PDI of the resveratrol nanoemulsion formulation were estimated to be 111.54 4.16 nm and 0.182 0.02, respectively. A typical representation of the globule size determination is shown in Figure 5. The nanoemulsions globule size and PDI matched those described in the literature [59].

#### 3.4.2. Zeta Potential

**Figure 6:**
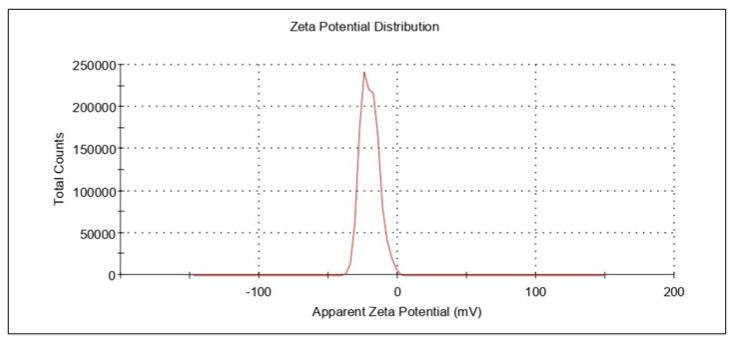
Representative plots for zeta potential.

The resveratrol nanoemulsion formulation’s zeta potential was measured as −40.96 ± 3.1 mV. (Figure 6). The zeta potential value of a system is considered a critical parameter since it permits or helps in the estimation of physical stability. The higher the zeta potential, either a positive value or a negative value, the greater the stability of that colloidal system. This is due to the formation of dispersions as a result of particle electrostatic repulsions. The electrostatic repulsions among globules with the same electrical charge prevent globule or droplet aggregation [45]. Thus, for the optimized formulation herein, the higher value of zeta potential ensured the stability of the developed nanoemulsion.

#### 3.4.3. Surface Morphology by TEM

**Figure 7:**
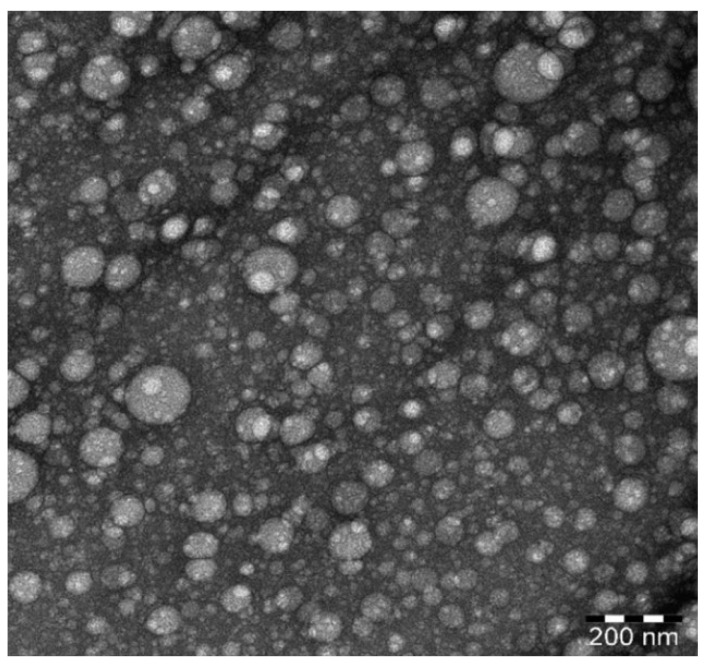
TEM image of the optimized formulation.

The optimized nanoemulsion TEM micrograph (Figure 7) exhibited a consistent spherical morphology with globule sizes around 100 nm. This result matched the globule size determined by the dynamic light scattering approach. Furthermore, the globule form was comparable to that of a previously described nanoemulsion generated by ultrasonication [40].

#### 3.4.4. Thermodynamic Stability Studies

Nanoemulsions are formulated with optimum concentrations of oil, water, and surfactant, and are thermodynamically stable, with no phase separation, cracking, or creaming. It is the thermostability that differentiates nanoemulsions from conventional emulsions, which have good kinetic stability but eventually phase separate with time. Thermodynamic stability studies ensure the stability of the optimized formulation [60,61,62]. The metastable formation can be detected by these tests. If the formulation is stable over the stressed conditions, we can ensure the avoidance of metastable formulations. Therefore, frequent tests need not be accomplished during the storage period, except when chemical reactions occur, such as pH variations or oxidation, which change the nature of the ingredients [43]. The results showed that the optimized formulation successfully survived all three stress tests. It was found that no creaming or separation occurred after the heating–cooling cycle, centrifugation, or freeze–thaw cycles. The result showed that optimized formulations were stable after heating–cooling cycles, freeze–thaw-cycles as well as centrifugation studies. Overall, the data here suggest that there was no phase separation observed after the thermodynamic stability studies.

### 3.5. Rheological Characterization

Nanoemulsion containing hydrogel was subjected to a temperature of 37 °C, and sol to gel transition was observed. At 25 °C, the viscosity was 198.1 ± 2.1 cps, and at 37 °C the viscosity increased to 377.6 ± 2.4 cps. The nanoemulsion hydrogel solution was analyzed for sol to gel transition behavior at 37 °C, and the results were in good agreement with Argenta et al. [38]. It has been claimed that the gelling process is reversible, and a sol–gel transition temperature exists (Tsol-gel). In general, Poloxamer 407 aqueous solutions remain fluid below Tsol-gel, but the solution becomes a semi-solid substance beyond this point. The possible reason for the thermogelation of poloxamer 407 could be because of the hydrophobic interactions of the copolymer chains, wherein these copolymer chains begin to combine into a micellar structure as the temperature rises. Indeed, the dehydration of the hydrophobic poly(propylene oxide) repeat units causes micelle formation, which is the first step in the gelation process [38].

### 3.6. In Vitro Drug Release

**Figure 8:**
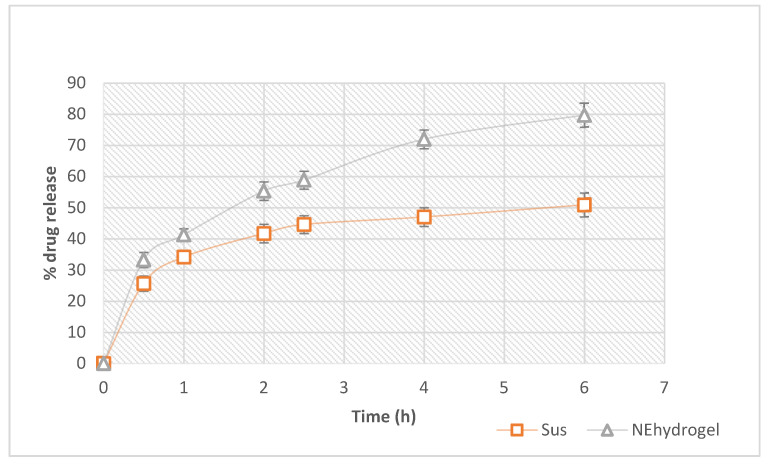
Comparison of release profiles of prepared nanoemulsion hydrogel and control suspension. NE hydrogel: nanoemulsion hydrogel; Sus: resveratrol suspension.

In comparison to the suspension, the resveratrol release from the optimized nanoemulsion hydrogel was considerably higher (Figure 8). The suspension formulation had a release of only 50.89 ± 2.47% after 6 h, whereas the optimized nanoemulsion hydrogel had a considerable release of nearly 80%. The reduced droplet size could potentially contribute to boosting resveratrol [63,64]. When the drug release from nanoemulsion hydrogel formulations was matched to that of a drug suspension, a significant difference (*p* < 0.001) was found. The tiny globule size of nanoemulsion formulations utilizing a high-energy approach can be linked to the percentage of resveratrol released from optimized nanoemulsion formulations. In the first 2.5 h, nearly 60% of the resveratrol in the hydrogel was released. This could be due to the nanoemulsion’s small droplet size, which provided a high surface area for drug release, and hence allowed a faster drug release. To check the formulation release, we used a dialysis bag with a pore size of 12,000 g/mol and a molecular cut-off of 12,000 g/mol. The optimized nano-formulation had differences in globule size, which could eventually yield an initial rapid release followed by a sustained release profile. Thus, drug release from the hydrogel matrix occurred in a sustained manner. This could help to maintain the drug contact time with the skin to produce a prolonged therapeutic effect. Hence, an instant release followed by the sustained release profile might be the result of varying size distribution as well as the particular pattern of release of the drug from the gel matrix. The tiny droplets cross rapidly through the gel matrix, and in turn, over the dialysis membrane pores, resulting in immediate release, while sustained release might result from drug liberation from the large droplets, which transport slowly across the hydrogel matrix, and thus also pass slowly through the pores of the dialysis membrane [53].

Thus, nanoemulsion hydrogel composed of poloxamer 407 from high molecular weight polymer chains has a higher density of intermolecular aggregates, and thus controls drug release more proficiently. This can also be linked to the higher viscosity and the uneven micelle distribution among more or less densely packed areas. Hence, pluronic 407, a triblock copolymer containing a central hydrophobic block of polypropylene glycol skirted by two hydrophilic blocks of polyethylene glycol chains, may represent a diffusional barrier to drug diffusion and thus proficiently control its release, establishing a promising delivery system for resveratrol [65].

### 3.7. Drug Release Kinetics

During in vitro release studies, the use of mathematical models can be an invaluable support for interpreting the drug diffusion process. Zero order, first order, Higuchi, and Korsmeyer–Peppas models are the common mathematical models used in drug release experiments for obtaining the one model which perfectly explains the kinetic release from the developed formulation. The best model will have a linear regression correlation coefficient close to 1. The results of regression parameters are shown in Table 5.

The zero-order kinetics models are the best fit for showing drug release from controlled release formulations, such as osmotic or coated polymer matrix systems. Here the drug diffusion rate is slower than that of the drug dissolution rate, creating a saturation state that allows a constant drug release rate. This is an ideal pattern for obtaining a prolonged pharmacological action. This effect is only applicable to formulations that do not disaggregate, and also to the same quantity of drug release/time unit. However, this pattern is unlikely to happen in practice, since it entails many limitations due to fewer adjustment factors for the model [46,66,67].

First-order kinetics symbolize a drug release profile proportional to the amount of drug in dosage form. Therefore, the quantity of drugs released will decrease with respect to time.

The Higuchi model defines the drug release based on the diffusion process as explained by Fick’s law of diffusion. Here, the release relates to the square root of time in h. This mechanism is mainly related to the release of drugs from the semisolid and solid matrix [67]. The Higuchi model is considered the most realistic model as compared to the first-order and zero-order models. Nevertheless, this equation does not consider phenomena such as swelling of the polymer or relaxation of the polymer matrix for drug transportation. However, this model can be best fitted for a poorly soluble one-dimensional matrix with no swelling capacity [46,67].

If the release follows a combination of Fickian transport and non-Fickian Case II transport, the semi-empirical model described by Korsmeyer et al. is the best choice. This model can be utilized when the release process is not exactly known, or if it is a combination of different independent processes. In this model, drug diffusion and hydrogel matrix relaxation are considered for analyzing the release mechanism. Thus, here, the structure of the matrix was taken into consideration. A value of n = 0.45 indicates Case-I (Fickian) diffusion or square root of time kinetics, 0.45 < n < 1 anomalous (non-Fickian) diffusion, n = 1, Case-II transport and n > 1 Super Case-II transport [68,69,70,71].

Here the resveratrol release from the nanoemulsion hydrogel followed the Higuchi model. From the values of release exponent (n), it is clear that the formulation showed Fickian release. The results indicate that the drug release from the hydrogel matrix was based on diffusion phenomena. This is in accordance with Fick’s first law of diffusion, which means that the drug release continues until a steady state is reached [72].

### 3.8. In Vitro Anticancer Activity on MCF-7 Breast Cancer Cell Lines

**Figure 9:**
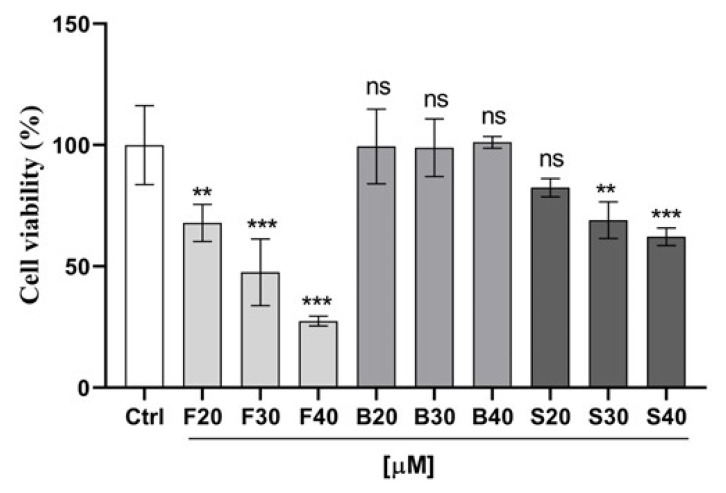
Effect of optimized formulation (F), suspension (S), blank (B), and control (C) on the cell viability of MCF-7 cancer cells after 24h treatment. Error bars are represented as mean ± SD (n = 4). Statistical analysis was performed using Turkey multiple comparisons (2-way ANOVA). *p* values are ** *p* > 0.01; *** *p* > 0.001 and ns < 0.05.

**Figure 10:**
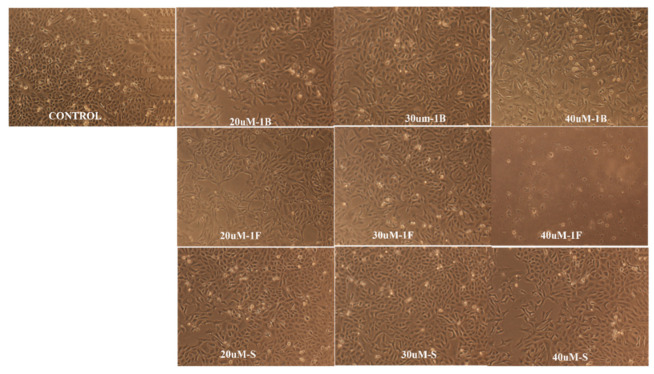
Morphological alterations of MCF7 cells after exposure to different formulations [optimized formulation (1F), suspension (S), blank (1B), and control] at different concentrations. Images were captured using a Nikon phase contrast microscope at ×10.

The impact of the optimized nanoemulsion on the viability of human breast cancer MCF-7 cells was evaluated utilizing MTT assay by comparing the optimized nanoemulsion with resveratrol suspension and blank formulation. As shown in Figure 9, both the nanoemulsion and suspension produced attrition of MCF-7 cells in a dose-dependent manner at a concentration ranging from 20 to 40 μM. To our surprise, the optimized nanoemulsion showed a very promising efficiency for inhibiting the growth of cancer cells at 40 μM with only 27% cell viability, followed by 30 µM and then 20 µM concentration of nanoemulsion with 48% and 68% viable cells. Additionally, the suspension resveratrol treatment, though significant (*p* < 0.05) in comparison to the control, showed less effect on cell viability compared to the nanoemulsion treatment. Approximately 62%, 69%, and 82% of viable cells were observed post-treatment with suspension resveratrol treatments at 40 µM, 30 µM, and 20 µM concentrations, respectively. Overall, our results from cell viability showed a greater effectiveness and proficiency of formulated nanoemulsion resveratrol compared to suspension resveratrol in the treatment of MCF7 cancer cells. Therefore, this showed that the formulated nanoemulsion resveratrol could be a more proficient strategy for cancer treatment. Graph (Figure 9) shows the activity of nanoemulsion, suspension, blank, and control on the cell viability of MCF-7 cancer cells in µM concentrations. Figure 10 shows the morphological alterations of MCF7 cells after exposure to different formulations at different concentrations. Micrograph images decrease cell survival and dead cells in the form of cell debris post-treatment with nanoemulsion and suspension resveratrol. Again, comparing nanoemulsion to suspension resveratrol treatment in MCF7, the images obtained from nanoemulsion treatment showed fewer viable cells relative to suspension treatment.

### 3.9. Storage Stability

**Figure 11:**
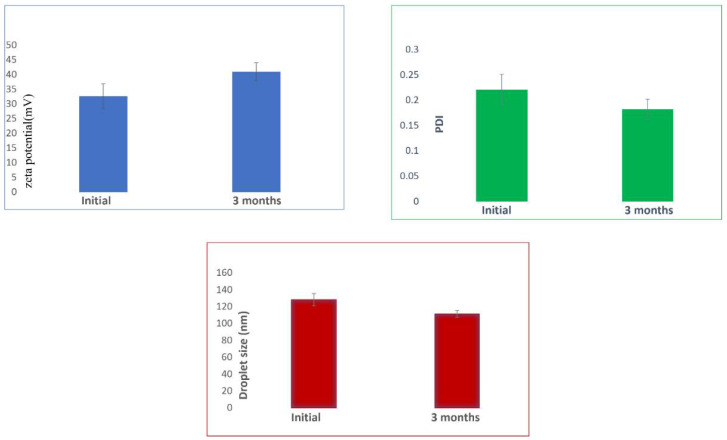
Stability assessment after storage of nanoemulsion at room temperature for three months.

The stability of the optimized nanoemulsion during the storage period of three months was assessed for droplet size, PDI, and zeta potential. It was noticed that even after 90 days of storage at room temperature, there was no significant difference in the results (Figure 11). It is reported that homogenization can extend storage stability by preventing creaming [73] and is critical in determining the size and possible stability. Thus, the data here signify that the homogenization process was able to provide adequate stability concerning droplet size, PDI, and zeta potential, which is also in agreement with an earlier study [74].

## 4. Conclusions

Implementing a central composite experimental design successfully enabled the optimization of resveratrol nanoemulsion with minimized droplet size and maximized zeta potential, which ensured maximum drug release as well as better stability of the optimized system. The small droplet size ensured maximum surface area and thereby better solubility, and the high charge guaranteed more electrostatic repulsion among the oil droplets for enhanced stability. The optimized nanoemulsion-loaded hydrogel proved to be effective in suppressing MCF-7 breast cancer cells. Our research demonstrated that the developed resveratrol nanoemulsion had a dose-dependent effect on the apoptosis of breast cancer cells; thus, we recommend consuming resveratrol-rich foods or supplements to help to prevent or manage breast cancer. In recent years, there has been significant focus on nanohydrogel-based local delivery of chemotherapeutics for breast cancer management, and the optimized nanoemulsion-loaded hydrogel formulation is a promising approach for managing breast cancer.

## Figures and Tables

**Table 1:** Independent variables and their levels.

Factor Code	Variables	Lower Level	Higher-Level
A	Speed (rpm)	6000	14,000
B	Time (min)	5	10
C	Surfactant concentration (%)	2	6

**Table 2:** Central composite design runs and their observed responses.

Run	Factor 1	Factor 2	Factor 3	Response 1	Response 2
	A: Speed (rpm)	B: Time (min)	C: Surfactant (%)	Droplet Size (nm)	Zeta Potential (mV)
1	14,000	5	6	172.1	26.5
2	6000	10	6	382.8	31.7
3	6000	10	2	337.4	41.5
4	10,000	7.5	4	151.6	40.3
5	14,000	5	2	464	37.1
6	10,000	5	4	162.8	42.3
7	6000	5	2	292.5	34.36
8	10,000	7.5	4	162.3	42.2
9	6000	7.5	4	147.2	38.4
10	10,000	7.5	4	162.4	42.2
11	10,000	10	4	184.45	41.4
12	14,000	10	2	302.45	34.9
13	6000	5	6	189.8	31.8
14	14,000	7.5	4	137.3	44.8
15	10,000	7.5	2	369.9	28.7
16	10,000	7.5	4	165.4	42.1
17	10,000	7.5	6	183.53	30.9
18	10,000	7.5	4	163.9	42.4
19	14,000	10	6	161.4	38.4

**Table 3:** ANOVA data for globule size of resveratrol nanoemulsion.

Source	Sum of Squares	df	Mean Square	*F*-Value	*p*-Value
Model	1.754 × 10^5^	11	15,947.81	323.22	<0.0001
A-Speed	1684.05	2	842.03	17.07	0.0020
B-Time	762.13	1	762.13	15.45	0.0057
C-Surfactant %	45,781.46	1	45,781.46	927.88	<0.0001
AB	21,038.85	2	10,519.43	213.20	<0.0001
AC	19,267.67	2	9633.84	195.25	<0.0001
BC	11,171.39	1	11,171.39	226.42	<0.0001
B^2^	984.69	1	984.69	19.96	0.0029
C^2^	40,718.05	1	40,718.05	825.26	<0.0001
Residual	345.38	7	49.34		
Lack of Fit	225.67	3	75.22	2.51	0.1972
Pure Error	119.71	4	29.93		
Cor Total	1.758 × 10^5^	18			

**Table 4:** ANOVA for zeta potential of resveratrol nanoemulsion.

Source	Sum of Squares	df	Mean Square	*F*-Value	*p*-Value
Model	996.26	11	90.57	78.43	<0.0001
A-Speed	96.06	2	48.03	41.59	0.0001
B-Time	12.45	1	12.45	10.78	0.0134
C-Surfactant %	9.49	1	9.49	8.21	0.0241
AB	111.07	2	55.54	48.09	<0.0001
AC	59.12	2	29.56	25.60	0.0006
BC	143.31	1	143.31	124.10	<0.0001
B^2^	0.9810	1	0.9810	0.8494	0.3874
C^2^	358.28	1	358.28	310.24	<0.0001
Residual	8.08	7	1.15		
Lack of Fit	5.07	3	1.69	2.25	0.2253
Pure Error	3.01	4	0.7530		
Cor Total	1004.34	18			

**Table 5:** Kinetics release parameters.

Mathematical Models	Nanoemulsion Hydrogel
Zero-order	0.8917
First-order	0.9027
Higuchi	0.9916
Korsmeyer–Peppas release exponent (n)	0.431

## Data Availability

Data are contained in the article.

## References

[1] Sung H., Ferlay J., Siegel R.L., Laversanne M., Soerjomataram I., Jemal A., Bray F. (2021). Global Cancer Statistics 2020: GLOBOCAN Estimates of Incidence and Mortality Worldwide for 36 Cancers in 185 Countries. CA Cancer J. Clin..

[2] Miele E., Spinelli G.P., Miele E., Tomao F., Tomao S. (2009). Albumin-bound formulation of paclitaxel (Abraxane ABI-007) in the treatment of breast cancer. Int. J. Nanomed..

[3] Catania A., Barrajón-Catalán E., Nicolosi S., Cicirata F., Micol V. (2013). Immunoliposome encapsulation increases cytotoxic activity and selectivity of curcumin and resveratrol against HER2 overexpressing human breast cancer cells. Breast Cancer Res. Treat..

[4] Andrade S., Ramalho M.J., Pereira M.D.C., Loureiro J.A. (2018). Resveratrol Brain Delivery for Neurological Disorders Prevention and Treatment. Front. Pharmacol..

[5] Ogle W.O., Speisman R.B., Ormerod B.K. (2013). Potential of treating age-related depression and cognitive decline with nutraceutical approaches: A mini-review. Gerontology.

[6] Koushki M., Amiri-Dashatan N., Ahmadi N., Abbaszadeh H.A., Rezaei-Tavirani M. (2018). Resveratrol: A miraculous natural compound for diseases treatment. Food Sci. Nutr..

[7] Galiniak S., Aebisher D., Bartusik-Aebisher D. (2019). Health benefits of resveratrol administration. Acta Biochim. Pol..

[8] Breuss J.M., Atanasov A.G., Uhrin P. (2019). Resveratrol and Its Effects on the Vascular System. Int. J. Mol. Sci..

[9] Hou C.Y., Tain Y.L., Yu H.R., Huang L.T. (2019). The Effects of Resveratrol in the Treatment of Metabolic Syndrome. Int. J. Mol. Sci..

[10] Meng T., Xiao D., Muhammed A., Deng J., Chen L., He J. (2021). Anti-Inflammatory Action and Mechanisms of Resveratrol. Molecules.

[11] Jang M., Cai L., Udeani G.O., Slowing K.V., Thomas C.F., Beecher C.W., Fong H.H., Farnsworth N.R., Kinghorn A.D., Mehta R.G. (1997). Cancer chemopreventive activity of resveratrol, a natural product derived from grapes. Science.

[12] Buhrmann C., Shayan P., Kraehe P., Popper B., Goel A., Shakibaei M. (2015). Resveratrol induces chemosensitization to 5-fluorouracil through up-regulation of intercellular junctions, Epithelial-to-mesenchymal transition and apoptosis in colorectal cancer. Biochem. Pharmacol..

[13] Alavi M., Farkhondeh T., Aschner M., Samarghandian S. (2021). Resveratrol mediates its anti-cancer effects by Nrf2 signaling pathway activation. Cancer Cell Int..

[14] Ren B., Kwah M.X., Liu C., Ma Z., Shanmugam M.K., Ding L., Xiang X., Ho P.C., Wang L., Ong P.S. (2021). Resveratrol for cancer therapy: Challenges and future perspectives. Cancer Lett..

[15] Espinoza J.L., Kurokawa Y., Takami A. (2019). Rationale for assessing the therapeutic potential of resveratrol in hematological malignancies. Blood Rev..

[16] Cottart C.H., Nivet-Antoine V., Beaudeux J.L. (2014). Review of recent data on the metabolism, biological effects, and toxicity of resveratrol in humans. Mol. Nutr. Food Res..

[17] Kapetanovic I.M., Muzzio M., Huang Z., Thompson T.N., McCormick D.L. (2011). Pharmacokinetics, oral bioavailability, and metabolic profile of resveratrol and its dimethylether analog, pterostilbene, in rats. Cancer Chemother. Pharmacol..

[18] Walle T., Hsieh F., DeLegge M.H., Oatis J.E., Walle U.K. (2004). High absorption but very low bioavailability of oral resveratrol in humans. Drug Metab. Dispos. Biol. Fate Chem..

[19] Intagliata S., Modica M.N., Santagati L.M., Montenegro L. (2019). Strategies to Improve Resveratrol Systemic and Topical Bioavailability: An Update. Antioxidants.

[20] Machado N.D., Fernández M.A., Díaz D.D. (2019). Recent Strategies in Resveratrol Delivery Systems. ChemPlusChem.

[21] Zhao Y.N., Cao Y.N., Sun J., Liang Z., Wu Q., Cui S.H., Zhi D.F., Guo S.T., Zhen Y.H., Zhang S.B. (2020). Anti-breast cancer activity of resveratrol encapsulated in liposomes. J. Mater. Chem. B.

[22] Ahmadi Z., Mohammadinejad R., Ashrafizadeh M. (2019). Drug delivery systems for resveratrol, a non-flavonoid polyphenol: Emerging evidence in last decades. J. Drug Deliv. Sci. Technol..

[23] Kotta S., Khan A.W., Pramod K., Ansari S.H., Sharma R.K., Ali J. (2012). Exploring oral nanoemulsions for bioavailability enhancement of poorly water-soluble drugs. Expert Opin. Drug Deliv..

[24] Gorain B., Choudhury H., Nair A.B., Dubey S.K., Kesharwani P. (2020). Theranostic application of nanoemulsions in chemotherapy. Drug Discov. Today.

[25] Zanesco-Fontes I., Silva A.C.L., da Silva P.B., Duarte J.L., Di Filippo L.D., Chorilli M., Cominetti M.R., Martin A. (2021). [10]-Gingerol-Loaded Nanoemulsion and its Biological Effects on Triple-Negative Breast Cancer Cells. AAPS PharmSciTech.

[26] Tarik Alhamdany A., Saeed A.M.H., Alaayedi M. (2021). Nanoemulsion and Solid Nanoemulsion for Improving Oral Delivery of a Breast Cancer Drug: Formulation, Evaluation, and a Comparison Study. Saudi Pharm. J. SPJ Off. Publ. Saudi Pharm. Soc..

[27] Han B., Wang T., Xue Z., Wen T., Lu L., Meng J., Liu J., Wu S., Yu J., Xu H. (2021). Elemene Nanoemulsion Inhibits Metastasis of Breast Cancer by ROS Scavenging. Int. J. Nanomed..

[28] Attari F., Hazim H., Zandi A., Mazarei Z., Rafati H. (2021). Circumventing paclitaxel resistance in breast cancer cells using a nanoemulsion system and determining its efficacy via an impedance biosensor. Analyst.

[29] Azani H., Homayouni Tabrizi M., Neamati A., Khadem F., Khatamian N. (2021). The Ferula Assa-foetida Essential Oil Nanoemulsion (FAEO-NE) as the Selective, Apoptotic, and Anti-Angiogenic Anticancer Compound in Human MCF-7 Breast Cancer Cells and Murine Mammary Tumor Models. Nutr. Cancer.

[30] De Souza R., Zahedi P., Moriyama E.H., Allen C.J., Wilson B.C., Piquette-Miller M. (2010). Continuous docetaxel chemotherapy improves therapeutic efficacy in murine models of ovarian cancer. Mol. Cancer Ther..

[31] Zahedi P., De Souza R., Piquette-Miller M., Allen C. (2009). Chitosan-phospholipid blend for sustained and localized delivery of docetaxel to the peritoneal cavity. Int. J. Pharm..

[32] Zarrintaj P., Ahmadi Z., Saeb M.R., Mozafari M. (2018). Poloxamer-based stimuli-responsive biomaterials. Mater. Today Proc..

[33] Guo D.D., Hong S.H., Jiang H.L., Kim J.H., Minai-Tehrani A., Kim J.E., Shin J.Y., Jiang T., Kim Y.K., Choi Y.J. (2012). Synergistic effects of Akt1 shRNA and paclitaxel-incorporated conjugated linoleic acid-coupled poloxamer thermosensitive hydrogel on breast cancer. Biomaterials.

[34] Chung C.K., García-Couce J., Campos Y., Kralisch D., Bierau K., Chan A., Ossendorp F., Cruz L.J. (2020). Doxorubicin Loaded Poloxamer Thermosensitive Hydrogels: Chemical, Pharmacological and Biological Evaluation. Molecules.

[35] Matei I., Ariciu A.-M., Popescu E.I., Mocanu S., Neculae A.V.F., Savonea F., Ionita G. (2022). Evaluation of the Accessibility of Molecules in Hydrogels Using a Scale of Spin Probes. Gels.

[36] Rajeshwari H.R., Dhamecha D., Jagwani S., Patil D., Hegde S., Potdar R., Metgud R., Jalalpure S., Roy S., Jadhav K. (2017). Formulation of thermoreversible gel of cranberry juice concentrate: Evaluation, biocompatibility studies and its antimicrobial activity against periodontal pathogens. Mater. Sci. Eng. C Mater. Biol. Appl..

[37] Pelegrino M.T., De Araujo Lima B., Do Nascimento M.H.M., Lombello C.B., Brocchi M., Seabra A.B. (2018). Biocompatible and Antibacterial Nitric Oxide-Releasing Pluronic F-127/Chitosan Hydrogel for Topical Applications. Polymers.

[38] Argenta D.F., Bernardo B.D.C., Chamorro A.F., Matos P.R., Caon T. (2021). Thermosensitive hydrogels for vaginal delivery of secnidazole as an approach to overcome the systemic side-effects of oral preparations. Eur. J. Pharm. Sci. Off. J. Eur. Fed. Pharm. Sci..

[39] Jacob S., Nair A.B., Shah J., Sreeharsha N., Gupta S., Shinu P. (2021). Emerging role of hydrogels in drug delivery systems, tissue engineering and wound management. Pharmaceutics.

[40] Nair A.B., Shah J., Al-Dhubiab B.E., Jacob S., Patel S.S., Venugopala K.N., Morsy M.A., Gupta S., Attimarad M., Sreeharsha N. (2021). Clarithromycin solid lipid nanoparticles for topical ocular therapy: Optimization, evaluation, and in vivo studies. Pharmaceutics.

[41] Jacob S., Nair A.B., Al-Dhubiab B.E. (2017). Preparation and evaluation of niosome gel containing acyclovir for enhanced dermal deposition. J. Liposome Res..

[42] Morsy M.A., Abdel-Latif R.G., Nair A.B., Venugopala K.N., Ahmed A.F., Elsewedy H.S., Shehata T.M. (2019). Preparation and evaluation of atorvastatin-loaded nanoemulgel on wound-healing efficacy. Pharmaceutics.

[43] Shafiq-un-Nabi S., Shakeel F., Talegaonkar S., Ali J., Baboota S., Ahuja A., Khar R.K., Ali M. (2007). Formulation development and optimization using nanoemulsion technique: A technical note. AAPS PharmSciTech.

[44] Ali H.H., Hussein A.A. (2017). Oral nanoemulsions of candesartan cilexetil: Formulation, characterization and in vitro drug release studies. Aaps Open.

[45] Shah J., Nair A.B., Jacob S., Patel R.K., Shah H., Shehata T.M., Morsy M.A. (2019). Nanoemulsion Based Vehicle for Effective Ocular Delivery of Moxifloxacin Using Experimental Design and Pharmacokinetic Study in Rabbits. Pharmaceutics.

[46] Barradas T.N., Senna J.P., Cardoso S.A., de Holanda E.S.K.G., Elias Mansur C.R. (2018). Formulation characterization and in vitro drug release of hydrogel-thickened nanoemulsions for topical delivery of 8-methoxypsoralen. Mater. Sci. Eng. C Mater. Biol. Appl..

[47] Nair A., Gupta R., Vasanti S. (2007). In vitro controlled release of alfuzosin hydrochloride using HPMC-based matrix tablets and its comparison with marketed product. Pharm. Dev. Technol..

[48] Wang Y., Liu Y., Tang T., Luo Y., Stevens M.F.G., Cheng X., Yang Y., Shi D., Zhang J., Bradshaw T.D. (2020). The antitumour activity of 2-(4-amino-3-methylphenyl)-5-fluorobenzothiazole in human gastric cancer models is mediated by AhR signalling. J. Cell. Mol. Med..

[49] Leong C.O., Suggitt M., Swaine D.J., Bibby M.C., Stevens M.F., Bradshaw T.D. (2004). In vitro, in vivo, and in silico analyses of the antitumor activity of 2-(4-amino-3-methylphenyl)-5-fluorobenzothiazoles. Mol. Cancer Ther..

[50] Walstra P. (1996). Emulsion stability. Encyclopedia of Emulsion Technology.

[51] Rallison J. (1984). The deformation of small viscous drops and bubbles in shear flows. Ann. Rev. Fluid Mech..

[52] Solans C., Izquierdo P., Nolla J., Azemar N., Garcia-Celma M.J. (2005). Nano-emulsions. Curr. Opin. Colloid Interface Sci..

[53] Kotta S., Khan A.W., Ansari S.H., Sharma R.K., Ali J. (2015). Formulation of nanoemulsion: A comparison between phase inversion composition method and high-pressure homogenization method. Drug Deliv..

[54] Parveen R., Baboota S., Ali J., Ahuja A., Vasudev S.S., Ahmad S. (2011). Oil based nanocarrier for improved oral delivery of silymarin: In vitro and in vivo studies. Int. J. Pharm..

[55] Akrawi S.H., Gorain B., Nair A.B., Choudhury H., Pandey M., Shah J.N., Venugopala K.N. (2020). Development and optimization of naringenin-loaded chitosan-coated nanoemulsion for topical therapy in wound healing. Pharmaceutics.

[56] Chen H., Chang X., Weng T., Zhao X., Gao Z., Yang Y., Xu H., Yang X. (2004). A study of microemulsion systems for transdermal delivery of triptolide. J. Control. Release Off. J. Control. Release Soc..

[57] Abdelkader H., Alani A.W., Alany R.G. (2014). Recent advances in non-ionic surfactant vesicles (niosomes): Self-assembly, fabrication, characterization, drug delivery applications and limitations. Drug Deliv..

[58] Shah H., Nair A.B., Shah J., Jacob S., Bharadia P., Haroun M. (2021). Proniosomal vesicles as an effective strategy to optimize naproxen transdermal delivery. J. Drug Deliv. Sci. Technol..

[59] Colombo M., Figueiró F., de Fraga Dias A., Teixeira H.F., Battastini A.M.O., Koester L.S. (2018). Kaempferol-loaded mucoadhesive nanoemulsion for intranasal administration reduces glioma growth in vitro. Int. J. Pharm..

[60] Shehata T.M., Elnahas H.M., Elsewedy H.S. (2022). Development, Characterization and Optimization of the Anti-Inflammatory Influence of Meloxicam Loaded into a Eucalyptus Oil-Based Nanoemulgel. Gels.

[61] Nawaz A., Latif M.S., Alnuwaiser M.A., Ullah S., Iqbal M., Alfatama M., Lim V. (2022). Synthesis and Characterization of Chitosan-Decorated Nanoemulsion Gel of 5-Fluorouracil for Topical Delivery. Gels.

[62] Bali V., Ali M., Ali J. (2010). Study of surfactant combinations and development of a novel nanoemulsion for minimising variations in bioavailability of ezetimibe. Colloids Surf. B Biointerfaces.

[63] Liu Y., Fu X., Lan N., Li S., Zhang J., Wang S., Li C., Shang Y., Huang T., Zhang L. (2014). Luteolin protects against high fat diet-induced cognitive deficits in obesity mice. Behav. Brain Res..

[64] Jaiswal M., Dudhe R., Sharma P.K. (2015). Nanoemulsion: An advanced mode of drug delivery system. 3 Biotech.

[65] Carteau D., Bassani D., Pianet I. (2008). The “Ouzo effect”: Following the spontaneous emulsification of trans-anethole in water by NMR. Comptes Rendus Chim..

[66] Feldstein M., Tohmakhchi V., Malkhazov L., Vasiliev A., Plate N. (1996). Hydrophilic polymeric matrices for enhanced transdermal drug delivery. Int. J. Pharm..

[67] Costa P., Sousa Lobo J.M. (2001). Modeling and comparison of dissolution profiles. Eur. J. Pharm. Sci. Off. J. Eur. Fed. Pharm. Sci..

[68] Peppas N.A. (2014). 1. Commentary on an exponential model for the analysis of drug delivery: Original research article: A simple equation for description of solute release: I II. Fickian and non-Fickian release from non-swellable devices in the form of slabs, spheres, cylinders or discs, 1987. J. Control. Release Off. J. Control. Release Soc..

[69] Korsmeyer R.W., Gurny R., Doelker E., Buri P., Peppas N.A. (1983). Mechanisms of solute release from porous hydrophilic polymers. Int. J. Pharm..

[70] Ritger P.L., Peppas N.A. (1987). A simple equation for description of solute release II. Fickian and anomalous release from swellable devices. J. Control. Release.

[71] Nair A.B., Chaudhary S., Shah H., Jacob S., Mewada V., Shinu P., Aldhubiab B., Sreeharsha N., Venugopala K.N., Attimarad M. (2022). Intranasal Delivery of Darunavir-Loaded Mucoadhesive In Situ Gel: Experimental Design, In Vitro Evaluation, and Pharmacokinetic Studies. Gels.

[72] Ikeda S., Nishinari K. (2001). “Weak gel”-type rheological properties of aqueous dispersions of nonaggregated kappa-carrageenan helices. J. Agric. Food Chem..

[73] Hidajat M.J., Jo W., Kim H., Noh J. (2020). Effective droplet size reduction and excellent stability of limonene nanoemulsion formed by high-pressure homogenizer. Colloids Interfaces.

[74] Gulati N., Kumar Chellappan D., Tambuwala M., Aljabali A.A.A., Prasher P., Kumar Singh S., Anand K., Sharma A., Kumar Jha N., Gupta G. (2021). Oral Nanoemulsion of Fenofibrate: Formulation, Characterization, and In Vitro Drug Release Studies. Assay Drug Dev. Technol..

